# Construction situation, costs and charges associated with pharmacy intravenous admixture services: multi-center cross-sectional survey based on 137 medical institutions in mainland China

**DOI:** 10.1186/s12913-020-05336-w

**Published:** 2020-06-24

**Authors:** Chunsong Yang, Bing Yao Kang, Lingli Zhang, Dan Yu

**Affiliations:** 1 Department of Pharmacy, Evidence-based Pharmacy Center, West China second hospital, Sichuan University. Key Laboratory of Birth Defects and Related Diseases of Women and Children (Sichuan University), Ministry of Education, Chengdu, China; 2 Department of Epidemiology, West China School of Public Health, and West China Fourth Hospital, Sichuan University, Chengdu, China; 3 Department of Pediatric clinic, West China second hospital, Sichuan University, Chengdu, China; 4 Department of Children’s Genetic Endocrinology and Metabolism, West China Second University Hospital, Sichuan University, Sichuan University, Chengdu, China

**Keywords:** PIVAS, Construction, Cost, Charge, Survey

## Abstract

**Background:**

To investigate the construction situation, costs and charges associated with pharmacy intravenous admixture services (PIVAS) to provide references for the construction and development of PIVAS in mainland China.

**Methods:**

A multi-center cross-sectional survey was conducted via a WeChat Group targeting PIVAS leaders in hospitals to investigate the basic situation of PIVAS, including opening time, area, number of PIVAS, equipment, management mode, PIVAS costs and charges, as well as numbers of beds, open wards, and staff, and analyze differences in PIVAS construction at different provincial and hospital levels.

**Results:**

137 questionnaires were collected from 29 provinces, representing a response rate of 99.3%. Most participants (88.4%) were from Level III Hospitals. The number of years of operations of PIVAS ranged from 1 to 22 (median: 6). PIVAS site area ranged between 100 and 1973 m^2^; daily average infusion volume was concentrated in the ranges 0–1000 bags (29.9%, 41/137) and 1001–2000 bags (26.3%, 36/137). In terms of PIVAS management mode, the vast majority used separate pharmacy management (65.0%, 89/137). Only 52.6% (72/137) of PIVAS have standardized charges, and 70.1% (96/137) operate at a loss. The median costs of mixed tumor chemotherapy drugs, total parenteral nutrition, general medicine, antibiotics were 20, 35, 4 and 5 RMB, respectively. With the exception of a few features, PIVAS construction does not obviously differ among different regions and hospital levels.

**Conclusions:**

In recent years, PIVAS in China has developed rapidly and become relatively large. The main problems are that most provinces lack standards for charges and PIVAS construction differs among hospitals. Therefore, standards for PIVAS construction and charges should be developed to provide a reference for the future development of PIVAS.

## Background

Pharmacy intravenous admixture services (PIVAS) is a medical department that integrates clinical pharmacy and scientific research according to international standards and good manufacturing practice (GMP) standards. In a clean operating environment, trained pharmacists or nurses in the PIVAS department conducted admixture of intravenous drugs according to operating procedures, including work with tumor chemotherapy drugs, total parenteral nutrition (TPN), general medicine and antibiotics. PIVAS also provided patients with rational drug use services [1, 2].

In 2002 and 2011, the “Regulations on the Administration of Pharmaceutical Affairs in Medical Institutions” published by the Ministry of Health of China clearly stated: medical institutions should establish PIVAS to achieve centralized admixture for tumor chemotherapy drugs and TPN. In 2010, the “Regulations on the Quality Management of Centralized Dispensing of Intravenous Drugs” clarified basic requirements and admixture procedures for PIVAS, marking the normalization of PIVAS in China [3].

In 1999, Shanghai Jing’an district central hospital established the first PIVAS in China. Other hospitals in China subsequently followed, and PIVAS now exists in more than 1100 hospitals [4]. With the continuous development of hospital and clinical pharmacies, PIVAS have significantly reduced patient morbidity and mortality caused by contaminated or incorrectly mixed intravenous infusions [5–7].

In 2016, Mi et al. [8] surveyed 97 PIVAS centers in Chinese hospitals to understand recent advances in PIVAS in China, but this cross-sectional study only included Level I and II hospitals, leaving the situation of Level III hospitals unclear. Additionally, 73% of the sample came from China’s eastern provinces, meaning the sample did not adequately reflect the status of PIVAS throughout China.

Therefore, we used a multi-center cross-sectional survey to investigate the construction situation, cost and charges of PIVAS to provide a reference for the construction and development of PIVAS in mainland China.

## Methods

### Study design

This study comprises a multi-center cross-sectional survey conducted from March 2019 to April 2019.

### Participants

PIVAS managers from each hospital were selected as survey respondents via the WeChat group of the Intravenous Dispensing Management and Application Branch of the China Medical Education Association, there are 300 members in the group.

### Data collection

This study used self-developed questionnaires to collect data and investigate the construction situation of China’s PIVAS. The contents of the questionnaire included: (1) basic participant information: region, level of their hospital, position; (2) basic situation of PIVAS: opening time, area, management mode, numbers of PIVAS, beds, open wards, equipment (biological safety cabinet, horizontal laminar flow table) and staff, and degree of recognition of doctors and nurses; (3) PIVAS costs and service charges: PIVAS service charge standard, intravenous admixture service costs, and profit and loss situation.

### Data analysis

If the data were normally distributed, they were expressed as x ± s, t test or analysis of variance was used. If the data were not normally distributed, the rank sum test was used. Categorical variables were analyzed by the chi-square test. The difference was statistically significant at *P* < 0.05. Data were analyzed using SPSS 21.0 software (IBM Corp., Armonk, NY, U.S.A).

## Result

### Basic characteristics of PIVAS (Tables 1 and 2)

With the help of the questionnaire platform, 138 questionnaires were collected, of which 137 were valid questionnaires, for a response rate of 46% (138/300) and the rate of valid questionnaire of 99.3% (137/138).

**Table 1:** Basic characteristics of PIVAS

Variable	Range (median)	Mean ± SD
The number of years of operations of PIVAS	1–22 years (6)	6.31 ± 3.88
Area (m^2^)	100–1973 (540)	582.22 ± 281.64
Number of open wards	1–134 (24)	26.83 ± 20.48
Number of biological safety cabinet	0–32 (6)	6.26 ± 3.72
Number of horizontal laminar flow table	0–27 (6)	7.23 ± 3.97
Number of pharmacists	1–110 (12)	17.18 ± 16.04
Number of nurses	4–52 (11.5)	13.46 ± 8.50
Number of workers	0–30 (3)	5.26 ± 5.46

**Table 2:** Basic characteristics of PIVAS

Variable		Percentage (n/total)/Mean ± SD
Region	Eastern China	56.2% (77/137)
	Western China	27.0% (37/137)
	Central China	16.8% (23/137)
Hospital level	Level III Grade A hospital	74.5% (102/137)
	Level III Grade B hospital	13.9% (19/137)
	Level II Grade A hospital	11.7% (16/137)
Number of PIVAS	1	81.8% (112/137)
	2	16.8% (23/137)
	≥3	1.4% (2/137)
Number of beds	0–500	22.6% (31/137)
	501–1000	27.0% (37/137)
	1001–1500	25.5% (35/137)
	1501–2000	13.9% (19/137)
	2001–2500	5.1% (7/137)
	≥2501	5.8% (8/137)
PIVAS daily average infusion volume (bag)	0–1000	29.9% (41/137)
	1001–2000	26.3% (36/137)
	2001–3000	13.9% (19/137)
	3001–4000	15.3% (21/137)
	≥4001	14.6% (20/137)
Management mode	Pharmacy management separately	65.0% (89/137)
	Pharmacist nurse co-manage	10.2% (14/137)
	Pharmacy-based, nursing department-assisted	24.8% (34/137)

The investigated PIVAS were located in 29 provinces, namely Anhui, Beijing, Chongqing, Fujian, Gansu, Guangdong, Guangxi, Guizhou, Hainan, Hebei, Heilongjiang, Henan, Hubei, Hunan, Jiangsu, Jiangxi, Jilin, Liaoning, Inner Mongolia, Qinghai, Shandong, Shanghai, Shanxi, Shaanxi, Sichuan, Tianjin, Xinjiang, Yunnan and Zhejiang. 56.2% (77/137) of PIVAS were located in eastern China, 27.0% (37/137) in western China, and 16.8% (23/137) in central China. The majority of PIVAS (74.5%) were located in Level III Grade A hospitals.

The number of years of operations of PIVAS ranged from 1 to 22 (median: 6 years). Moreover, the area of PIVAS ranged between 100 and 1973 m^2^, with a median of 540 m^2^. The number of open wards ranged from 1 to 134, with a median of 24. In terms of PIVAS equipment, the mean number of biological safety cabinets is six, and the mean number of horizontal laminar flow tables is seven. Finally, the median numbers of pharmacists, nurses and workers were 12, 11.5 and 3, respectively.

81.8% (112/137) of the hospitals surveyed had opened 1 PIVAS, while the remainder (22.6%) has opened two or more PIVAS. In terms of scale, the largest group of PIVAS in the sample (27.0%, 37/137) provided service for 501–1000 beds, with the next largest group (25.5%, 35/137) serving 1501–1500 beds. Daily average infusion volume was mainly concentrated in the bands 0–1000 bags (29.9%, 41/137) and 1001–2000 bags (26.3%, 36/137). In terms of management mode, the vast majority of PIVAS (65.0%, 89/137) used separate pharmacy management.

### The operations, charges and cost of PIVAS service (Tables 3 and 4)

Only 52.6% (72/137) of PIVAS have charge standards, and 70.1% (96/137) operate at a loss. Most doctors (67.4%, 88/137) and nurses (86.8%, 119/137) recognized the performance of PIVAS, but based on labor results only 14.6% (20/137) of PIVAS were reflected in salary performance at the hospital level. The median costs of admix tumor chemotherapy drugs, TPN, general medicine and antibiotics were 20, 35, 4 and 5 RMB, respectively.

**Table 3:** the operations and charges of PIVAS

Variable		Percentage (n/total)/Mean ± SD
PIVAS charge standard	Yes	52.6% (72/137)
	No	47.4% (65/137)
PIVAS profit and loss situation	Profit	7.3% (10/137)
	Loss	70.1% (96/137)
	Balance	22.6% (31/137)
The degree of recognition of the doctors	Very disagree	0% (0/137)
	Less agree	4.4% (6/137)
	General agree	31.4% (43/137)
	Slightly agree	46.0% (63/137)
	Very agree	18.2% (25/137)
The degree of recognition of the nurses	Very disagree	0% (0/137)
	Less agree	3.6% (5/137)
	General agree	9.5% (13/137)
	Slightly agree	47.4% (65/137)
	Very agree	39.4% (54/137)
The labor results of PIVAS are reflected in the performance of the hospital level?	Not reflected	48.2% (66/137)
	General	37.2% (51/137)
	Reflected	14.6% (20/137)

**Table 4:** The cost of intravenous admixture service (yuan/bag) (including consumables, labor, depreciation of fixed assets)

Variable	Range (median)	Mean ± SD
Tumor chemotherapy drugs	1–200 (20)	28.10 ± 25.13
Total Parenteral Nutrition, TPN	1–150 (35)	37.39 ± 23.84
General medicine	1–50 (4)	6.84 ± 8.08
Antibiotics	1–50 (5)	9.02 ± 10.34

### Subgroup analysis (Tables 5 and 6)

In the subgroup analysis of different regions, we found only that the number of biological safety cabinets was higher in the eastern and central regions than in the western region (χ^2^ = 4.355, *P* = 0.015).

**Table 5:** Basic characteristics of PIVAS in different region

Variable		Eastern China	Western China	Central China	***x***^**2**^/F	P
PIVAS daily average infusion volume (bag)	0–1000	17	19	5	10.937	0.205
	1001–2000	22	7	7		
	2001–3000	13	3	3		
	3001–4000	13	4	4		
	≥4001	12	4	4		
Management mode	Pharmacy management separately	47	27	15	3.657	0.454
	Pharmacist nurse co-manage	8	2	4		
	Pharmacy-based, nursing department-assisted	22	8	4		
The number of years of operations of PIVAS		6.5 ± 3.94	6.5 ± 4.54	5.4 ± 2.15	0.807	0.668
Area (m^2^)		611 ± 276.21	539 ± 281.16	556 ± 301.29	0.922	0.400
Number of open wards		28.3 ± 22.06	21.2 ± 16.01	31.1 ± 20.30	2.133	0.122
Number of biological safety cabinet		6.8 ± 4.07	4.8 ± 2.84	6.8 ± 3.18	4.355	0.015*
Number of horizontal laminar flow table		7.5 ± 3.54	6.2 ± 3.83	7.9 ± 5.27	1.669	0.192
Number of pharmacists		17.6 ± 16.20	15.4 ± 15.17	18.8 ± 17.28	0.359	0.699
Number of nurses		143 ± 10.46	13.2 ± 6.96	11.6 ± 6.37	0.284	0.754

*: *P* < 0.05

**Table 6:** Basic characteristics of PIVAS in different level of hospital

Variable		Level III Grade A Hospital	Level III Grade B Hospital	Level II Grade A Hospital	***x***^**2**^/t	P
PIVAS daily average infusion volume (bag)	0–1000	31	4	5	21.468	0.006*
	1001–2000	22	5	9		
	2001–3000	15	3	1		
	3001–4000	15	6	0		
	≥4001	19	1	0		
Management mode	Pharmacy management separately	72	9	8	7.457	0.114
	Pharmacist nurse co-manage	7	3	4		
	Pharmacy-based, nursing department-assisted	23	7	4		
The number of years of operations of PIVAS		6.6 ± 3.87	5.0 ± 2.67	6.1 ± 4.96	1.403	0.249
Area (m^2^)		604.7 ± 297.3	588.9 ± 241.83	431.1 ± 160.67	2.700	0.071
Number of open wards		29.9 ± 21.86	21.1 ± 14.09	14.2 ± 8.56	11.726	0.003*
Number of biological safety cabinet		6.6 ± 4.00	6.2 ± 2.67	4.0 ± 1.71	3.613	0.030*
Number of horizontal laminar flow table		7.8 ± 4.26	6.8 ± 2.50	4.3 ± 1.40	16.664	0.000*
Number of pharmacists		19.3 ± 16.97	13.9 ± 13.14	7.4 ± 6.26	18.588	0.000*
Number of nurses		13.8 ± 6.62	7.3 ± 2.14	18.0 ± 15.82	7.111	0.029*

*: *P* < 0.05

In the subgroup analysis of different levels of hospital, we found significant differences in daily average infusion volume (χ^2^ = 21.468, *P* = 0.006), and numbers of open wards (χ^2^ = 11.726, *P* = 0.003), biological safety cabinets (χ^2^ = 3.613, *P* = 0.030), horizontal laminar flow tables (χ^2^ = 16.664, *P* = 0.000), pharmacists (χ^2^ = 18.588, *P* = 0.000) and nurses (χ^2^ = 7.111, *P* = 0.029).

## Discussion

To our knowledge, this was the first and largest cross-sectional study to investigate the construction situation, costs and charges of PIVAS in China. A total of 137 hospitals located in China’s eastern, western and central regions completed this survey, and so the research results are representative of the current status of PIVAS construction in China. Generally, PIVAS developed rapidly in China and are relatively large, reflected in larger site areas, numbers of open wards, quantities of equipment, and daily average infusion volumes.

However, PIVAS in China are associated with many problems: (1) a unified management model is lacking, with the vast majority of PIVAS in China being separately managed by pharmacies, perhaps because most belonged to the pharmacy departments of their associated hospitals. However, two other management modes existed, meaning the advantages and disadvantages of different management models should be clarified to better control the quality of PIVAS operations; (2) Most provinces lack standards for charges, and most PIVAS operated at a loss, but many leaders in PIVAS thought the results of their labor were not reflected in hospital level salary performance, making it necessary to develop standards for PIVAS charges to motivate staff and highlight the value of their work. (3) The cost of admixing four kinds of medicine differed considerably among different PIVAS, perhaps because cost calculation methods differed among hospitals, highlighting the importance of developing unified standards for cost calculation.

Additionally, we analyze the relationship between PIVAS construction status for different provincial and hospital levels, and found no regional differences except in number of biological safety cabinets. This result showed similarity in development speed and scale between different regions, and suggested that national policies may contribute to the PIVAS development. Particularly, the publication of the “Regulations on the Quality Management of Centralized Dispensing of Intravenous Drugs” may promote PIVAS construction or development. We also found that different levels of hospital differed significantly in daily average infusion volume, as well as numbers of open wards, equipment, and staff. This result was intuitive, with higher level hospitals being larger, and having more equipment, larger infusion volumes, and more numerous staff.

This study has some limitations. Firstly, this study did not use random sampling to select participants. Naturally it is difficult to conduct a cross-sectional study using randomization given the absence of accurate numbers or clear listing of PIVAS in China. However, the study participants were drawn from 29 Chinese provinces, meaning the results are reasonably representative. Secondly, cross-sectional study could not make causal inferences, so further prospective research is needed to track the future development of PIVAS.

## Conclusion

The development of PIVAS in China has been fast and PIVAS in China are also relatively large. The main problems are the lack of charge standards in most provinces and differences in PIVAS construction among hospitals in China. Therefore, it is necessary to develop standards for PIVAS construction and charges to provide a reference for the future development of PIVAS.

## Data Availability

The datasets generated and/or analyzed during the current study are not publicly available because they are subject to the West China Second University Hospital, Sichuan University. However, the data and materials are available from the corresponding author on reasonable request.

## Abbreviations

PIVAS: Pharmacy intravenous admixture services
TPN: Total parenteral nutrition
GMP: Good manufacturing practice
